# Filtered Cathodic Vacuum Arc Deposition for Inkjet-Printed OLED Encapsulation

**DOI:** 10.3390/ma19030638

**Published:** 2026-02-06

**Authors:** Zhuo Gao, Songju Li, Lei Wang, Lin Chen, Xianwen Sun, Dong Fu

**Affiliations:** 1 State Key Laboratory of Luminescent Materials and Devices, Institute of Polymer Optoelectronic Materials and Devices, South China University of Technology, Guangzhou 510640, China; 2 Guangdong Juhua Printed Display Technology Co., Ltd., Guangzhou 510700, China; songju.li@tcl.com (S.L.); sunxw@tcl.com (X.S.); fud@tcl.com (D.F.); 3 College of Nuclear Science and Technology, Beijing Normal University, Beijing 100875, China; linchen@bnu.edu.cn

**Keywords:** inkjet printing, filtered cathodic vacuum arc deposition, thin-film encapsulation, reliability

## Abstract

To improve the low deposition rate of atomic layer deposition (ALD), we introduced filtered cathodic vacuum arc (FCVA) technology for the high-rate deposition of Al_2_O_3_ films. The FCVA-Al_2_O_3_ process achieved a deposition rate of 15 nm/min, which is approximately an order of magnitude higher than that of conventional ALD. This process does not involve hydrogen, preventing hydrogen ion penetration and thereby ensuring the high stability of the oxide TFT backplane. FCVA-Al_2_O_3_ films were integrated with inkjet-printed (IJP) organic layers to form a hybrid thin-film encapsulation (TFE) structure for OLEDs. The resulting laminated encapsulation exhibited excellent water vapor barrier properties (WVTR, Water Vapor Transmission Rate of 1.2 × 10^−4^ g/m^2^/day), demonstrating the great potential of FCVA for packaging high-throughput and high-performance flexible electronics. In addition to evaluating barrier properties (surface roughness, residual stress, and WVTR) to assess the suitability of TFE, the impact of FCVA technology was assessed via oxide thin-film transistor (TFT) electrical performance and OLED device reliability tests. The electrical properties of oxide TFTs show no significant degradation post-encapsulation, while OLED performance, despite a slight increase in current efficiency, remains effectively unchanged. Additionally, the lifetime of OLED devices reached 300 h under accelerated aging conditions (85 °C, 85% relative humidity), which is nearly twice that of devices without FCVA-Al_2_O_3_ encapsulation.

## 1. Introduction

As display technologies have developed, OLED technology has become widely used in various fields. Benefiting from features such as self-emission, high contrast, a wide viewing angle, fast response, low power consumption, ultra-thin design, and a flexible display, various kinds of OLED display products are emerging [1]. In particular, flexible OLED products such as curved monitors, foldable mobile phones, and rollable TVs are very popular [2]. Inkjet printing is one of the most promising manufacturing technologies for next-generation flexible OLED display technologies due to its advantages of a high material utilization rate, low cost, and ease of large-area production. However, challenges regarding the lifetime and performance stability of printed OLED devices still remain, which are one of the key factors hindering their large-scale mass production [3,4]. To accelerate the development of flexible display technology, it is necessary to not only synthesize new types of organic light-emitting materials with high performance and a long lifetime but also develop efficient TFE technology, with good anti-bending and encapsulation performance [5,6]. TFE technology protects flexible OLED devices by alternately forming a laminated structure with organic and inorganic films, which can effectively reduce the thickness of the device and ensure the flexibility of OLED devices. Compared with traditional glass encapsulation, it is more suitable for ultra-thin and ultra-light flexible OLED displays [7].

In the early stages, K. Yamashita’s research group from Nagoya University in Japan fabricated polymer encapsulation films using plasma-enhanced chemical vapor deposition (PECVD), which increased the lifetime of OLED devices by four times [8]. A. Heeger and others from the University of California, Santa Barbara, United States, fabricated a water vapor barrier layer with the use of spin-coating Cytop^TM^ (a perchlorinated polymer), which increased the half-life of the devices by more than five times [9]. In Germany, Riedl’s research group developed Al_2_O_3_/ZrO_2_ nano-laminated vapor and oxygen barrier layer technology using ALD technology, which is able to significantly eliminate pinholes and surface defects in the encapsulation film and reduce the corrosion of Al_2_O_3_. The Al_2_O_3_ layer deposited using ALD was dense and smooth, and a WVTR of 4.7 × 10^−5^ g/m^2^/day was achieved with a thickness of only 130 nm under 70% relative humidity and 70 °C conditions [10]. Vitex Systems in the United States developed Barix^TM^ encapsulation technology, which forms a barrier to prevent water vapor and oxygen from penetrating into flexible OLED devices by alternately depositing acrylic resin monomers and inorganic films. The performance of the Barix^TM^ barrier layer can be adjusted by changing the number of layers and the composition of the acrylic resin and inorganic layers in the film coating. The Barix^TM^ film had a transmittance of more than 80% in the visible-light range, a surface roughness of less than 10 nm, and a WVTR of 10^−4^~10^−6^ g/m^2^/day [11].

ALD technology sequentially introduces precursors into the reaction chamber, enabling uniform adsorption and simultaneous reaction on the substrate surface to form atomic-scale bonds [12]. This cyclic process ensures the growth of one atomic layer per cycle, resulting in highly uniform and dense thin films [13,14,15]. The method achieves precise nanometer-scale control over the thickness of the barrier that effectively blocks water vapor and oxygen [16,17]. However, ALD-Al_2_O_3_ suffers from low deposition rates, and hydrogen incorporation during the process is unavoidable. As a common impurity in oxide TFTs, hydrogen ions (both positive and negative) can accumulate in the channel region, degrading device performance and electrical reliability [18,19,20].

FCVA technology generates plasma through the evaporation of a metal cathode via discharge. The cathode material ions fly along the magnetic filter duct, finally arrive at the surface of the substrate, and then react with oxygen to form metal oxides [21]. Since the magnetic filter duct can filter out large particle droplets and other impurities, what reaches the substrate is a high-purity and high-concentration plasma of the cathode material [22,23]. Compared with ALD, FCVA enables faster, large-area deposition of thin films with excellent step coverage, compactness, and adjustable stress. Notably, the hydrogen-free nature of FCVA-Al_2_O_3_ helps to preserve the stability of oxide TFTs, while its low-temperature processing ensures compatibility with temperature-sensitive materials such as those used in IJP OLEDs [24,25,26,27,28].

The primary objective of this study is to overcome the intrinsic low-speed limitation of ALD by employing the FCVA technique. We aim to (1) achieve a high deposition rate of dense Al_2_O_3_ films; (2) integrate them with IJP organic films to form a robust laminated TFE structure; and (3) validate the effectiveness of this novel encapsulation approach by assessing the stability of the encapsulated oxide TFT and the reliability of the encapsulated IJP OLED devices.

## 2. Experiment

### 2.1. Deposition of Al_2_O_3_ Film Using FCVA

In contrast to ALD, the FCVA process for depositing Al_2_O_3_ operates without the need for a hydrogen element. Under vacuum conditions, an intense arc discharge is initiated between the cathode and anode, generating extremely high-temperature cathode spots on the cathode surface. The movement of these arc spots is systematically controlled across the aluminum target using a scanning coil to ensure uniform consumption of the cathode material. The electrons, atoms, photons, and micron particles of cathode material are abundantly emitted from the arc spot. At high pressure and density, the electrons and atoms undergo intense collisions, forming a highly ionized, high-density plasma of the cathode material. Guided by the magnetic field, electrons transport the aluminum plasma through a bent duct to the substrate where it is reacted with oxygen, resulting in the formation of Al_2_O_3_. In contrast, uncharged particles like large droplets and neutral particles adhere to the inside walls of the magnetic filter duct, and these are removed [29,30].

**Figure 1:**
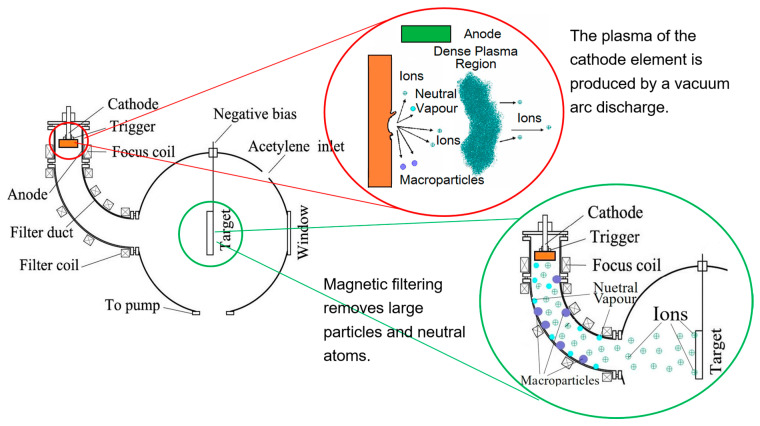
Schematic diagram of magnetic filter cathode vacuum arc deposition.

The FCVA deposition system comprises components such as a vacuum chamber, a high-voltage pulse power supply, a magnetic-filtering curved duct, focusing coils, filtering coils, and a substrate holder. Through equipment modifications and process optimization, the deposition rate of the Al_2_O_3_ films can be adjusted within the range of 10 to 20 nm/min. The FCVA deposition process is schematically illustrated in Figure 1.

First, the PEN (DuPont, Wilmington, DE, USA) sheets and silicon wafers were ultrasonically cleaned sequentially in acetone, ethanol, and deionized water, followed by drying with nitrogen gas to eliminate surface particles that could compromise film density.

FCVA-Al_2_O_3_ films were deposited using an Al target as the cathode source. Prior to deposition, the vacuum chamber was evacuated to a base pressure of 4.0 × 10^−3^ Pa and heated to a working temperature of 80 °C. The cathode was then activated using an arc current of 95 A. During the deposition process, a gas mixture consisting of 6 sccm argon (Ar) and 16 sccm oxygen (O_2_) was continuously introduced into the chamber. The distance between the target and the substrate was fixed at 260 mm. Meanwhile, a negative bias was applied to the substrate, with a frequency of 50 Hz and a voltage of 5 kV. Under these conditions, the deposition rate of Al_2_O_3_ was approximately 15 nm/min, and all fabricated films exhibited a thickness of approximately 100 nm.

ALD-Al_2_O_3_ films were deposited on both PEN and silicon wafer at 85 °C, using trimethylaluminum (TMA) as the precursor and plasma O_2_ as the reactant. TMA was relatively stable and readily available, meaning that it could be adsorbed onto a variety of substrates of different materials. Compared with O_3_ and H_2_O, using the plasma O_2_ as the reactant can produce films with better barrier properties and reduce deposition time. The final 100 nm Al_2_O_3_ film was deposited in 720 cycles, resulting in a growth per cycle (GPC) of ~0.14 nm/cycle.

The resulting films were then characterized for their key properties, including surface roughness, residual stress, WVTR, and visible-light transmittance.

### 2.2. Deposition of Al_2_O_3_ Films on Oxide TFT Backplanes

**Figure 2:**
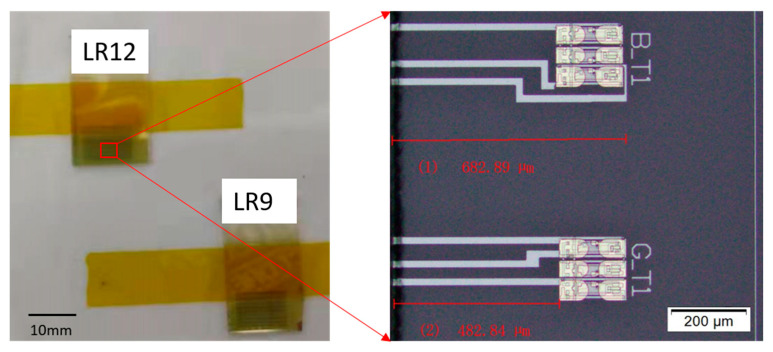
Schematic diagram of the TFT test structure.

The dies (10 mm × 20 mm) of the Test Element Group (TEG) were diced from the oxide TFT backplane and mounted onto a glass substrate (100 mm × 100 mm) using thermally stable tape. To assess the positional effects, one die was placed in the central region and another at the edge region of the substrate. During the deposition of the Al_2_O_3_ film via the FCVA process, the substrate was rotated at a constant speed to improve film thickness uniformity. The oxide TFT samples are illustrated in Figure 2.

The electrical characteristics of the oxide TFTs, such as threshold voltage (Vth), carrier mobility, and subthreshold swing (SS), were compared before and after the deposition of the Al_2_O_3_ films to analyze the influence of the FCVA process on the TFT’s performance. With this analysis, we aimed to determine whether the high-energy plasma directly damages the oxide TFTs during deposition and whether the absence of hydrogen in the reaction contributes to enhanced electrical stability.

### 2.3. Thin-Film Encapsulation of IJP OLED Devices

A multilayer thin-film encapsulation structure was fabricated on 50 mm × 50 mm inkjet-printed OLED devices (self-fabricated) to evaluate the practical effectiveness of Al_2_O_3_ films deposited using FCVA technology for OLED encapsulation applications.

A silicon oxynitride (SiO_x_N_y_) layer was deposited as the first inorganic barrier on the IJP OLED devices via plasma-enhanced chemical vapor deposition (PECVD). The samples were then transferred to an inkjet printing system, where an acrylic resin ink was deposited onto the SiO_x_N_y_ film via inkjet printing. The ink used was a UV-curable polymer (SDI, Yongin, Republic of Korea) solution based on poly(methyl methacrylate) (PMMA). The printed liquid film was allowed to level out through static settling. Subsequently, the film was exposed to 395 nm of ultraviolet (UV) light, initiating photo polymerization of the acrylic monomers via a photo initiator, which resulted in crosslinking and solidification into a solid film as the organic planarization layer. The thickness of the organic layer was 12 μm; the thickness variation was controlled within ±3.0%. This organic layer exhibits excellent conformality and flexibility, effectively encapsulating particles and filling voids while also serving to modulate the stress within the multilayer structure, thereby improving encapsulation reliability.

**Figure 3:**
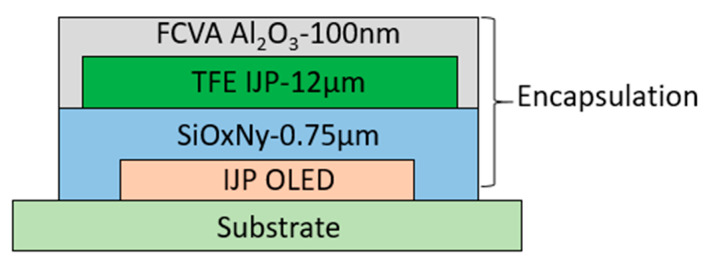
Schematic diagram of IJP OLED stack structure encapsulation.

Finally, a third inorganic barrier layer of Al_2_O_3_ was deposited atop the organic buffer layer using FCVA technology, thereby completing the multilayer TFE structure (FCVA-Al_2_O_3_ (100 nm)/TFE IJP (12 µm)/SiO_x_N_y_ (0.75 µm)). A schematic of the encapsulated IJP OLED device structure is presented in Figure 3.

The optoelectronic performance of the IJP OLED devices, including parameters such as current, voltage, luminance, and efficiency, was compared before and after TFE was applied to analyze the influence of the FCVA-Al_2_O_3_ film on device performance. Additionally, the reference sample was encapsulated with a different structure (TFE IJP (12 µm)/SiO_x_N_y_ (0.75 µm)), without the FCVA-Al_2_O_3_ film. A comparative study was conducted to evaluate the operational lifetime and reliability between devices encapsulated using these two different methods under high-temperature and high-humidity conditions.

## 3. Results and Discussion

### 3.1. Characterization of Al_2_O_3_ Films Prepared Using FCVA

**Figure 4:**
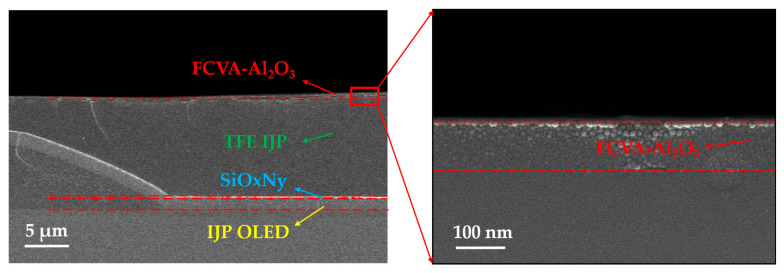
The cross-sectional SEM images of the multilayer TFE structure.

In this study, a 100 nm thick Al_2_O_3_ film was deposited via FCVA. This process took approximately 6–7 min, corresponding to a deposition rate of 15 nm/min. For comparison, depositing another Al_2_O_3_ film of the same thickness via ALD required about 20 min, with a deposition rate of less than 5 nm/min. FCVA-Al_2_O_3_ was applied in IJP OLED TFE, of which the multilayer structure was confirmed using a scanning electron microscope (SEM, Apreo 2 SEM, Thermo Fisher Scientific, Waltham, MA, USA). The layer thicknesses were as follows: the FCVA-Al_2_O_3_ film was approximately 100 nm; the TFE IJP film was 12 μm; and the SiO_x_N_y_ film was 0.75 μm. Cross-sectional SEM images of the multilayer TFE structure are shown in Figure 4.

**Figure 5:**
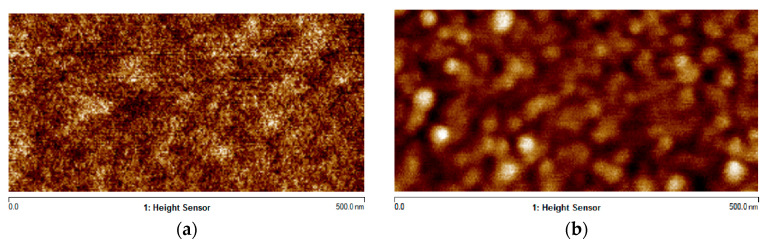
The surface roughness of Al_2_O_3_ films prepared using FCVA (**a**) and ALD (**b**).

The surface morphology and roughness of the films were characterized via Atomic Force Microscopy (AFM) over a scan area of 500 nm × 500 nm, using a Bruker Dimension Icon system (Bruker Corporation, Greater Boston, MA, USA) in tapping mode with a silicon cantilever. The Al_2_O_3_ film prepared using FCVA showed an average surface roughness (Ra) of 0.111 nm, while that deposited by ALD exhibited an Ra of 0.314 nm. Although both films displayed favorable surface morphology, the FCVA-deposited film demonstrated superior smoothness, as illustrated in Figure 5.

When fabricating Al_2_O_3_ films of identical thicknesses, the FCVA technique is able to achieve a deposition rate three times faster than ALD and yield a superior average surface roughness. This results in not only significantly reduced process time but also enhanced surface flatness.

The residual stress was measured using a stress measurement system (FSM-100, Frontier Semiconductor, Milpitas, CA, USA) with a 780 nm measuring laser. The curvature was measured before and after TFE on a 4-inch silicon wafer [31]. The results indicated that the residual stress of the Al_2_O_3_ film prepared using FCVA could be adjusted within a range of −140.4 MPa to −55.1 MPa. When combined with the IJP organic buffer layer, this film effectively modulates the overall stress distribution within the multilayer encapsulation structure. This stress engineering prevents issues such as cracking or peeling caused by stress mismatch, thereby significantly improving the encapsulation reliability and flexural endurance of the device.

The film thickness and refractive index were measured using an ellipsometer (SE-3, Raditech, Shanghai, China), and the optical transparence was measured with a UV/Vis spectrometer (LAMBDA 365, PerkinElmer, Waltham, MA, USA) with a scan wavelength ranging from 400 to 700 nm. Both films exhibited comparable refractive indices and visible-light transmittance, resulting in almost no impact on the light extraction of the OLED devices. Furthermore, the refractive index can be tailored during fabrication to minimize the potential impact. A detailed performance comparison between Al_2_O_3_ films prepared using FCVA and ALD is summarized in Table 1.

**Figure 6:**
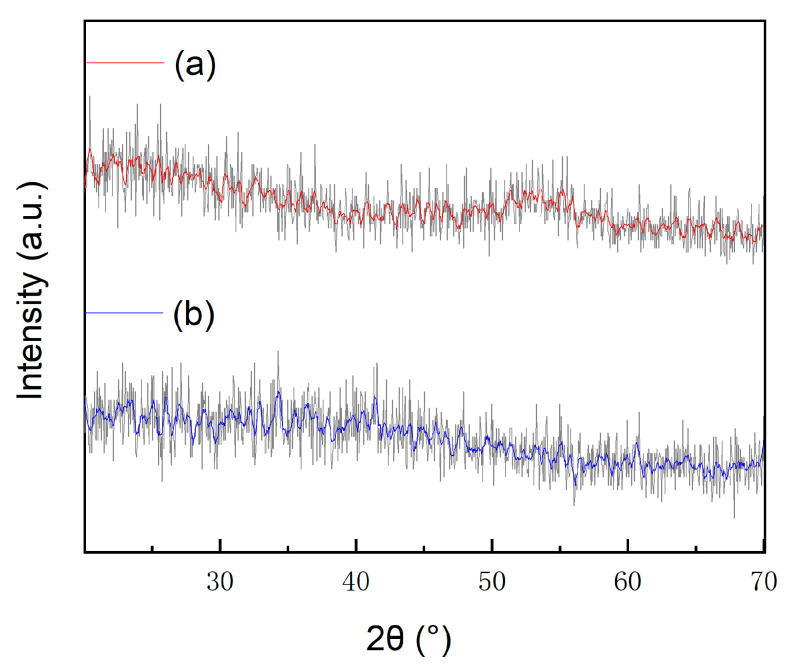
The XRD pattern of ALD-Al_2_O_3_ (a) and FCVA-Al_2_O_3_ (b).

The phase composition and structural nature of the Al_2_O_3_ films were analyzed using an X-ray diffractometer (XRD) measurement system (Empyrean, PANalytical, Almelo, The Netherlands). The XRD measurements were performed on films deposited on silicon wafers, scanning a 2θ range from 30° to 70° with a step size of 0.05°. The XRD patterns of both ALD-Al_2_O_3_ and FCVA-Al_2_O_3_ showed no distinct characteristic peaks, indicating that both films possess an amorphous structure, as shown in Figure 6. Compared with ZrO_2_, TiO_2_, and MgO, which were prone to crystallizing, non-crystallized Al_2_O_3_ did not contain any grain boundaries that tend to form a channel for water vapor or oxygen.

**Figure 7:**
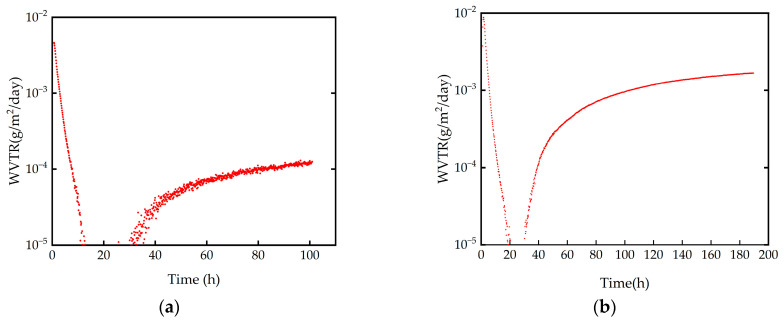
The chart of WVTR of Al_2_O_3_ films prepared using FCVA (**a**) and ALD (**b**).

The WVTR was measured using a moisture measurement system (CELASIS, Moresco, Kobe, Japan) based on precise element inspection with a mass spectrometer. The measuring samples were prepared via depositing a thin film on the PEN substrate with a thickness of 125 μm. All samples in this study were measured in an environment of 40 °C and 90% relative humidity. The WVTR testing exhibited values of 1.2 × 10^−4^ g/m^2^/day for the Al_2_O_3_ film deposited using FCVA compared with 1.6 × 10^−3^ g/m^2^/day for its ALD-prepared counterpart. Both films exhibited barrier properties as good as those achieved using single-layer encapsulation; notably, the FCVA-fabricated Al_2_O_3_ film demonstrated a WVTR one order of magnitude lower, indicating significantly superior water vapor barrier performance. These results are summarized in Figure 7.

Although the Al_2_O_3_ film prepared using FCVA exhibited good water vapor barrier properties, its performance still fell short of the requirements of OLED applications by approximately an order of magnitude. Therefore, it must be combined with an inkjet-printed organic buffer layer to form a multilayer TFE structure, which is essential for achieving the necessary WVTR performance [32].

### 3.2. Stability of Oxide TFT Encapsulated with FCVA-Al_2_O_3_

**Figure 8:**
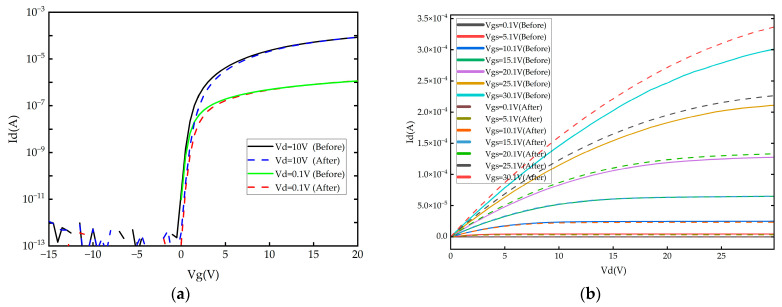
The transfer curves (**a**) and the output curves (**b**) of the same TFT before and after encapsulation with FCVA-Al_2_O_3_.

To further verify the feasibility of applying Al_2_O_3_ thin films prepared using FCVA technology in TFE, the electrical properties of the same oxide TFT device were measured before and after TFE using the B1500A analyzer (Keysight Technologies, Santa Rosa, CA, USA). The transfer characteristics were measured with gate voltages (Vgs) of −15 V to 20 V at drain voltages (Vd) of 0.1 V and 10 V. The output characteristics were obtained by increasing Vd from 0 V to 30 V at Vgs of 0.1, 5.1, 10.1, 15.1, 20.1, 25.1, and 30.1 V. The transfer and output curves of the TFTs before and after encapsulation with FCVA-Al_2_O_3_ are shown in Figure 8.

As shown in Table 2, the electrical properties of the oxide TFT were consistent before and after encapsulation with FCVA-Al_2_O_3_. Specifically, the encapsulation resulted in a marginal shift in Vth (from 1.08 V to 1.10 V), a negligible change in mobility (from 14.02 to 14.31 cm^2^/V·s), and a slight improvement in SS (from 0.16 to 0.15 V/dec). These slight variations in TFT electrical characteristics were considered within the testing error range. It is well-established that hydrogen incorporation is a primary source of instability in oxide TFTs, typically leading to significant negative Vth shifts. The absence of such degradation strongly suggests that use of the FCVA-Al_2_O_3_ process does not introduce detrimental hydrogen-related species.

It is postulated that the inherent low-energy plasma in the FCVA-Al_2_O_3_ process causes a negligible impact on TFT electrical properties. In contrast to Al_2_O_3_ films prepared using ALD or SiN_x_ films prepared using CVD, the FCVA process does not involve a hydrogen element [33,34]; the hydrogen element is one of the most common impurities in oxide TFTs, and it can reside in the channel as positive or negative ions, influencing the hole concentration and consequently affecting the TFT’s performance and electrical stability [35,36,37,38].

### 3.3. Reliability of IJP OLED Encapsulated with FCVA-Al_2_O_3_

**Figure 9:**
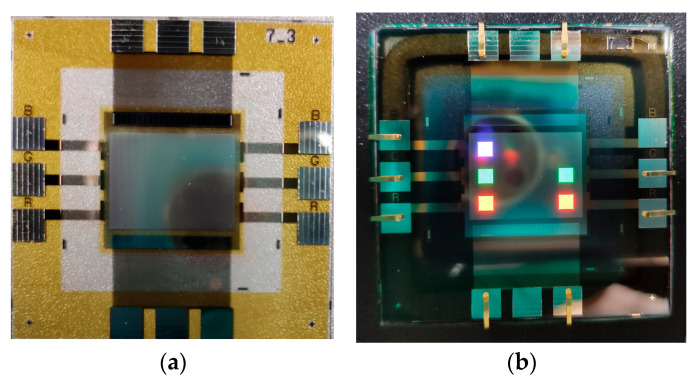
The diagram of IJP OLED device (**a**) and lighting test (**b**).

In this study, IJP OLED devices (self-fabricated) were selected as samples to verify the encapsulation reliability of the Al_2_O_3_ film. The device dimensions were 50 mm × 50 mm, each containing five independent emission pixels, as shown in Figure 9. A tandem encapsulation structure was formed by applying an FCVA-prepared Al_2_O_3_ film combined with an IJP organic buffer layer (self-fabricated). The optoelectronic properties of the OLED devices were measured before and after encapsulation using an optoelectronic performance analyzer (FS-5500GA4, Fstar, Suzhou, China). The bias voltage was applied using a Keithley 2400 DC power supply (Keithley Instruments, Solon, OH, USA), with an increase from −1 V to 8 V at 0.2 V increments to collect current-voltage characteristics. Device luminance, color coordinates, and spectral data were measured with a CS2000 spectrometer (Konica Minolta, Tokyo, Japan).

**Figure 10:**
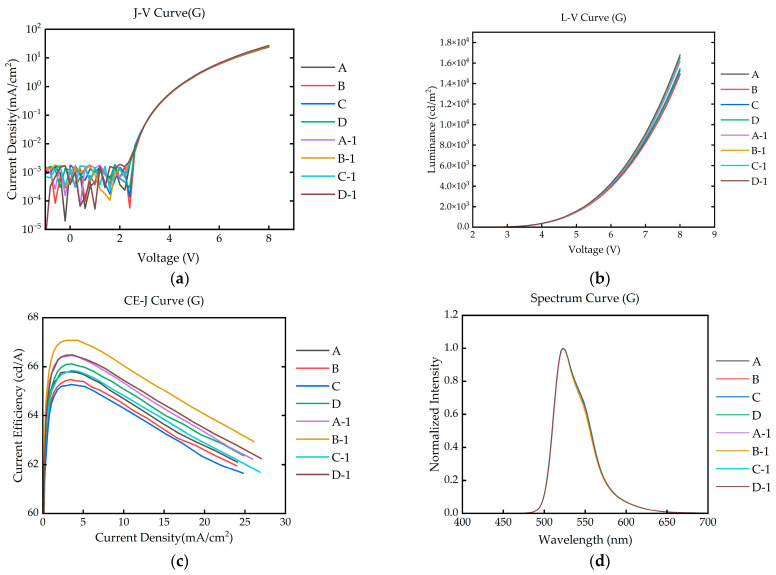
The IJP OLED performance comparison before and after encapsulation using FCVA: J-V curve (**a**), L-V curve (**b**), CE-J curve (**c**), and EL spectrum curve (**d**). A/B/C/D represent the OLED performance before encapsulation, and A-1/B-1/C-1/D-1 represent the OLED performance after encapsulation.

The optoelectronic performances of the IJP OLED devices before and after encapsulation with the FCVA-Al_2_O_3_ film are presented in Figure 10. For the devices (A, B, C, D) with the same device structure and process flow, the current density–voltage (J-V), luminance–voltage (L-V), and electroluminescence (EL) spectrum curves showed excellent consistency before and after encapsulation. Although the current efficiency (CE-J) curves exhibited minor shifts post-encapsulation, the results collectively indicate that the FCVA Al_2_O_3_ film did not cause a performance degradation; rather, it contributed to a certain improvement in the light out-coupling efficiency.

The IJP OLED devices underwent reliability testing in a controlled-environment chamber (MHK-408QK, Terchy, Suzhou, China) under accelerated aging conditions (85 °C, 85% relative humidity). Electroluminescence from a fixed emission region was periodically recorded while maintaining a constant driving voltage across the device.

A shrinkage of the OLED emission area was observed after 150 h in the reference sample, and this increased to four dark spots after 230 h; the largest one almost spanned the entire emission area. Therefore, we speculate that the lifetime of the reference sample rapidly declined within 80 h, and the TFE completely failed. In contrast, the first dark spot was observed after 300 h in the sample encapsulated with the FCVA-Al_2_O_3_ layer. Relative to the reference, the test sample showed a 150 h delay in the initial appearance of dark spots and a two-fold increase in the overall lifetime. The results are summarized in Table 3. Dark spots indicating shrinkage of emission area are highlighted with red circles.

The reliability test results demonstrate that the application of the FCVA-Al_2_O_3_ film for OLED encapsulation could delay the shrinkage of the OLED pixel emission area, thereby effectively prolonging the device’s operational lifetime under adverse conditions.

## 4. Conclusions

Compared with ALD-Al_2_O_3_, FCVA-Al_2_O_3_ has a faster deposition rate and lower WVTR, which is a more promising barrier, and it can lead to substantial gains in throughput and efficiency. A critical consideration is its compatibility with oxide TFT and IJP OLED. Due to its hydrogen-free nature, the FCVA-Al_2_O_3_ process effectively prevents any degradation of the electrical stability in oxide TFTs.

The key optoelectronic performance of IJP OLED devices, encapsulated with the FCVA-Al_2_O_3_ film, remains highly consistent post-encapsulation, and the lifetime of these devices is nearly double that of devices not encapsulated with FCVA-Al_2_O_3_. Undoubtedly, applying FCVA to the TFE of IJP OLEDs is an effective strategy to enable the extension of device lifespan without compromising optoelectronic performance.

The application of FCVA-Al_2_O_3_ for IJP OLED TFE represents a novel application of FCVA technology in the display field and shows considerable promise. Nevertheless, several aspects of this study merit further investigation to deepen our understanding. These include a more detailed analysis of hydrogen content and film quality in FCVA-Al_2_O_3_ films, a comprehensive investigation of oxide TFT bias stress stability, and a systematic evaluation of the LT_50_ lifetime of the encapsulated OLED devices.

Furthermore, the industrial-scale deployment of FCVA technology, especially for large-area and flexible display manufacturing, requires a system redesign focusing on multisource configurations and linear scanning mechanisms to achieve the necessary deposition area and film uniformity.

## Figures and Tables

**Table 1:** Comparison of properties of Al_2_O_3_ films prepared using FCVA and ALD.

Properties	FCVA-Al_2_O_3_	ALD-Al_2_O_3_
Film Thickness (nm)	106.1	106.5
Average Roughness (nm)	0.111	0.314
Residual Stress (MPa)	−70	70
Refractive Index	1.684	1.632
Visible-Light Transmittance (%)	98.4	98
Water Vapor Transmission Rate (g/m^2^/day)	1.2 × 10^−4^	1.6 × 10^−3^

**Table 2:** Influence of FCVA technology on the electrical properties of oxide TFT.

Items	TFT Properties	Before Encapsulation	After Encapsulation
Encapsulated with CVD-SiN_x_	Vth (V)	0.53	0.54
	Mobility (cm^2^/Vs)	11.42	15.92
	SS (V/dec)	0.10	0.15
Encapsulated with FCVA-Al_2_O_3_	Vth (V)	1.08	1.10
	Mobility (cm^2^/Vs)	14.02	14.31
	SS (V/dec)	0.16	0.15

**Table 3:** Reliability test of IJP OLED encapsulated with FCVA-Al_2_O_3_.

TFE Structure	RA 0 h	RA 24 h	RA 150 h	RA 230 h	RA 300 h
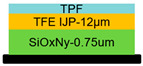	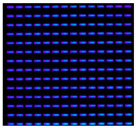	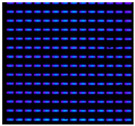	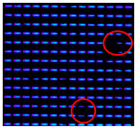	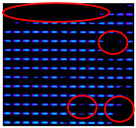	NA
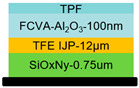	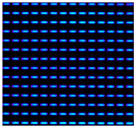	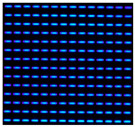	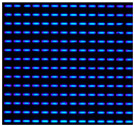	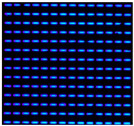	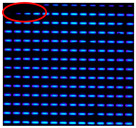

## Data Availability

The original contributions presented in this study are included in the article. Further inquiries can be directed to the corresponding authors.

## References

[1] Lee J.G., Gao Z., Fu D., Yan X. (2022). Flexible Printed OLED TV has Potential to Create a New Application Market. Inf. Disp..

[2] Jang H.J., Lee J.Y., Baek G.W., Kwak J., Park J.H. (2022). Progress in the development of the display performance of AR, VR, QLED and OLED devices in recent years. J. Inf. Disp..

[3] Park J.H., Jeong D.Y., Han K.Y. (2025). A Study on Improving Color Uniformity of Flexible OLEDs through Pixel-Define Layer Optimization in Inkjet Printing. ACS Appl. Electron. Mater..

[4] Hidehiro Y., Shuhei N., Takashi I., Yukiya U., Futoshi O. (2024). Mura-free G8.5 220ppi inkjet printing technology for OLED and QLED display panels. J. Soc. Inf. Disp..

[5] Jeong E.G., Kwon J.H., Kang K.S., Jeong S.Y., Choi K.C. (2020). A review of highly reliable flexible encapsulation technologies towards rollable and foldable OLEDs. J. Inf. Disp..

[6] Kwon B.H., Joo C.W., Cho H., Kang C., Yang J.H., Shin J.W., Kim G.H., Choi S., Nam S., Kim K. (2021). Organic/Inorganic Hybrid Thin-Film Encapsulation Using Inkjet Printing and PEALD for Industrial Large-Area Process Suitability and Flexible OLED Application. ACS Appl. Mater. Interfaces.

[7] Lee S., Han J.H., Lee S.H., Baek G.H., Park J.S. (2019). Review of organic/inorganic thin film encapsulation by atomic layer deposition for a flexible OLED display. J. Miner. Met. Mater. Soc..

[8] Granstrom J., Swensen J.S., Moon J.S., Rowell G., Yuen J., Heeger A.J. (2008). Encapsulation of organic light-emitting devices using a perfluorinated polymer. Appl. Phys. Lett..

[9] Meyer J., Görrn P., Bertram F., Hamwi S., Winkler T., Johannes H., Weimann T., Hinze P., Riedl T., Kowalsky W. (2009). Al_2_O_3_/ZrO_2_ nanolaminates as ultrahigh gas-diffusion barriers-a strategy for reliable encapsulation of organic electronics. Adv. Mater..

[10] Wei Y.W., Liu Z.C., Chen S.L. (2011). Optical characteristics of TiO_2_/Al_2_ O_3_ thin films and their atomic layer depositions. Chin. Opt..

[11] Kim H.K., Kim M.S., Kang J.W., Kim J.-J., Yi M.-S. (2007). High-quality thin-film passivation by catalyzer-enhanced chemical vapor deposition for organic light-emitting diodes. Appl. Phys. Lett..

[12] Steven M.G. (2010). Atomic layer deposition: An overview. Chem. Rev..

[13] Zhang B., Wang Z., Wang J., Chen X. (2024). Recent Achievements for Flexible Encapsulation Films Based on Atomic/Molecular Layer Deposition. Micromachines.

[14] Yang Y.Q., Duan Y., Chen P. (2014). Deposition of Al_2_O_3_ Film Using Atomic Layer Deposition Method at Low Temperature as Encapsulation Layer for OLEDs. Chin. J. Lumin..

[15] Li Y., Xiong Y.F., Yang H.Z., Cao K., Chen R. (2020). Thin film encapsulation for the organic light-emitting diodes display via atomic layer deposition. J. Mater. Res..

[16] Willis S.A., McGuinness E.K., Li Y., Losego M.D. (2021). Re-examination of the Aqueous Stability of Atomic Layer Deposited (ALD) Amorphous Alumina (Al_2_O_3_) Thin Films and the Use of a Postdeposition Air Plasma Anneal to Enhance Stability. Langmuir.

[17] Ylivaara O.M.E., Kilpi L., Liu X.W., Sintonen S., Ali S., Laitinen M., Julin J., Haimi E., Sajavaara T., Lipsanen H. (2017). Aluminum oxide/titanium dioxide nanolaminates grown by atomic layer deposition: Growth and mechanical properties. J. Vac. Sci. Technol. A.

[18] Shao Y., Ding S.J. (2018). Effects of hydrogen impurities on performances and electrical reliabilities of indium-gallium-zinc oxide thin film transistors. Acta Phys. Sin..

[19] Chen C., Cheng K.-C., Chagarov E., Kanicki J. (2011). Crystalline In–Ga–Zn–O Density of States and Energy Band Structure Calculation Using Density Function Theory. J. Appl. Phys..

[20] Hwang E.S., Kim J.S., Jeon S.M., Lee S.J., Jang Y., Cho D.-Y., Hwang C.S. (2018). In_2_Ga_2_ZnO_7_ oxide semiconductor-based charge trap device for NAND flash memory. Nanotechnology.

[21] Yuan H., Li Q., Yan W., Zhang Y., Chen L., Pan P., Luo J., Liao B., Ouyang X. (2022). A novel and efficient technology of depositing Al_2_O_3_ film for OLEDs thin film encapsulation. Vacuum.

[22] Mohapatra S., Oh M.S. (2025). Evaluating the Tribological Properties and Residual Stress of TiCrN Thin Films Deposited by Cathodic-Arc Physical Vapor Deposition Technique. Appl. Sci..

[23] Zhang B., Zhang L., Wu S., Peng X., Ouyang X., Liao B., Zhang X. (2025). A Study on the Structure and Properties of NiCr-DLC Films Prepared by Filtered Cathodic Vacuum Arc Deposition. Coatings.

[24] Kim E., Han Y., Kim W., Choi K.C., Im H.G., Bae B.S. (2013). Thin film encapsulation for organic light emitting diodes using a multi-barrier composed of MgO prepared by atomic layer deposition and hybrid materials. Org. Electron..

[25] Seo S.W., Jung E., Chae H., Cho S.M. (2012). Optimization of Al_2_O_3_/ZrO_2_ nanolaminate structure for thin-film encapsulation of OLEDs. Org. Electron..

[26] Kim L.H., Kim K., Park S., Jeong Y.J., Kim H., Chung D.S., Kim S.H., Park C.E. (2014). Al_2_O_3_/TiO_2_ nanolaminate thin film encapsulation for organic thin film transistors via plasma-enhanced atomic layer deposition. ACS Appl. Mater. Interfaces.

[27] Choi D.W., Kim S.J., Lee J.H., Chung K.B., Park J.S. (2012). A study of thin film encapsulation on polymer substrate using low temperature hybrid ZnO/Al_2_O_3_ layers atomic layer deposition. Curr. Appl. Phys..

[28] Martin P., Bendavid A. (2001). Review of the filtered vacuum arc process and materials deposition. Thin Solid Film..

[29] Yuan H., Zhang Y.F., Yan W.Q., Zhang Z.Q., Li Q., Chen L., Yin Z.Y., Liao B., Ouyang X.P., Ouyang X. (2022). Flexible alumina films prepared using high-bias pulse power for OLED thin film encapsulation. Ceram. Int..

[30] Yuan H., Zhang Y.F., Li Q., Yan W.Q., Zhang X., Ouyang X., Ouyang X.P., Chen L., Liao B. (2023). A Study of Al_2_O_3_/MgO Composite Films Deposited by FCVA for Thin-Film Encapsulation. Materials.

[31] Li S., Li M., Lan L., Fu D., Sun X., Gao Z. (2025). Comprehensive Investigation on the Stability of Silicon Nitride/Oxynitride as Thin-Film Encapsulation Layers Prepared by Plasma-Enhanced Chemical Vapor Deposition. ACS Appl. Mater. Interfaces.

[32] Gao S.Y., Kong X.-Z., Zhang F.-H., Lv L. (2012). Research Progress of Thin Film Encapsulation of Organic Light-Emitting Devices. J. Liq. Cryst. Disp..

[33] Jang J.T., Ko D., Choi S.J., Kim D.M., Kim D.H. (2021). Observation of Hydrogen-Related Defect in Subgap Density of States and Its Effects Under Positive Bias Stress in Amorphous InGaZnO TFT. IEEE Electron Device Lett..

[34] Ahn I.S., Ju B.K., Choi S.-H. (2024). Effects of Hydrogen Doping on a-GIZO Thin-Film Transistors with Hafnium Dioxide Gate Insulators Formed by Atomic Layer Deposition at Different Temperatures. IEEE Trans. Electron Devices.

[35] Nakashima M. (2014). Origin of major donor states in In–Ga–Zn oxide. J. Appl. Phys..

[36] Li Y., Liu Z., Jiang K., Hu X. (2013). H_2_ annealing effect on the structural and electrical properties of amorphous InGaZnO films for thin film transistors. J. Non-Cryst. Solids.

[37] Sallis S., Butler K.T., Quackenbush N.F., Williams D.S., Junda M., Fischer D.A., Woicik J.C., Podraza N.J., White B.E., Walsh A. (2014). Origin of deep subgap states in amorphous indium gallium zinc oxide: Chemically disordered coordination of oxygens. Appl. Phys. Lett..

[38] Kim J.Y., Kim H., Kim D., Jang H.W. (2025). Advancements of Amorphous IGZO-Based Transistors: Materials, Processing, and Devices. ACS Appl. Electron. Mater..

